# An Evolutionary Approach to the History of Barley (*Hordeum vulgare*) Cultivation in the Canary Islands

**DOI:** 10.1007/s10437-020-09415-5

**Published:** 2020-10-02

**Authors:** Jenny Hagenblad, Jacob Morales

**Affiliations:** 1 IFM Biology, Linköping University, SE-581 83 Linköping, Sweden; 2 Department of Historical Sciences, University of Las Palmas de Gran Canaria, Pérez del Toro 1, 35003 Las Palmas de Gran Canaria, Spain

**Keywords:** Barley (*Hordeum vulgare*), Canary Islands, Agricultural history, Plant cultivation, Evolution, ABC modeling

## Abstract

The Canary Islands are an archipelago that lies about 100 km west of North Africa. Barley (*Hordeum vulgare*) has been continuously cultivated since the colonization of the islands. To investigate the agricultural history of the islands, the DNA from multiple individuals of six extant landraces of barley was sequenced, and the resulting data were analyzed with ABC modeling. Estimates of separation times of barley populations on the different islands and the mainland were congruent with archaeological dating of the earliest settlements on the islands. The results of the genetic analyses were consistent with the continuous cultivation of barley on Lanzarote island since it was first colonized, but suggested cultivation was carried out at a smaller scale than on Gran Canaria and Tenerife. Contrary to archaeological evidence and early written historical sources, the genetic analyses suggest that barley was cultivated on a larger scale on Tenerife than on Gran Canaria. The genetic analysis of contemporary barley added support to the dating of the colonization of the islands and pointed to the need for more archaeological data concerning barley cultivation on Tenerife.

**Electronic supplementary material:**

The online version of this article (10.1007/s10437-020-09415-5) contains supplementary material, which is available to authorized users.

## Résumé

Les îles de Canaries est un archipel situé à environ 100 km à l’ouest de l'Afrique du Nord. L'orge (*Hordeum vulgare*) a été cultivée continuellement sur les îles depuis leur première colonisation. Pour étudier l'histoire agricole des îles, l'ADN de plusieurs individus de six variétés locales contemporaines d'orge a été séquencé et les données résultantes ont été analysées avec la modélisation ABC. Les estimations des temps de séparation des populations d'orge sur les différentes îles et sur le continent correspondaient à la datation archéologique de la colonisation des îles. La cultivation continue d'orge sur l’île de Lanzarote depuis sa colonisation a été soutenu, mais à une échelle plus réduite que celle de Gran Canaria et de Tenerife. Contrairement aux preuves archéologiques et aux premières sources historiques écrites, les analyses génétiques suggèrent que l’orge a été cultivée à une plus grande échelle à Tenerife qu'à Gran Canaria. L'analyse génétique de l'orge contemporaine a confirmé la datation de la colonisation des îles, mais a également souligné la nécessité de disposer de plus de données archéologiques concernant la cultivation de l'orge à Tenerife.

## Introduction

The history of agricultural species is, in many cases, inseparable from the history of humans, and genetic analysis of extant agricultural species can serve to further the understanding of past agricultural societies. Such analyses can, for example, inform the degree of continuity in cultivation, the comparative scale of agricultural practices, and the timing of the colonization of an area. These topics are central to understanding the cultural history of a region. Insights from genetic studies can be particularly indispensable in situations where archaeological data are insufficient or lacking. In other cases, genetic analysis of extant specimens can serve as a useful complement to archaeological investigations. In this study, we use extant barley to improve our understanding of the colonization and agricultural history of the Canary Islands.

### Pre-Hispanic History of the Canary Islands

**Fig. 1:**
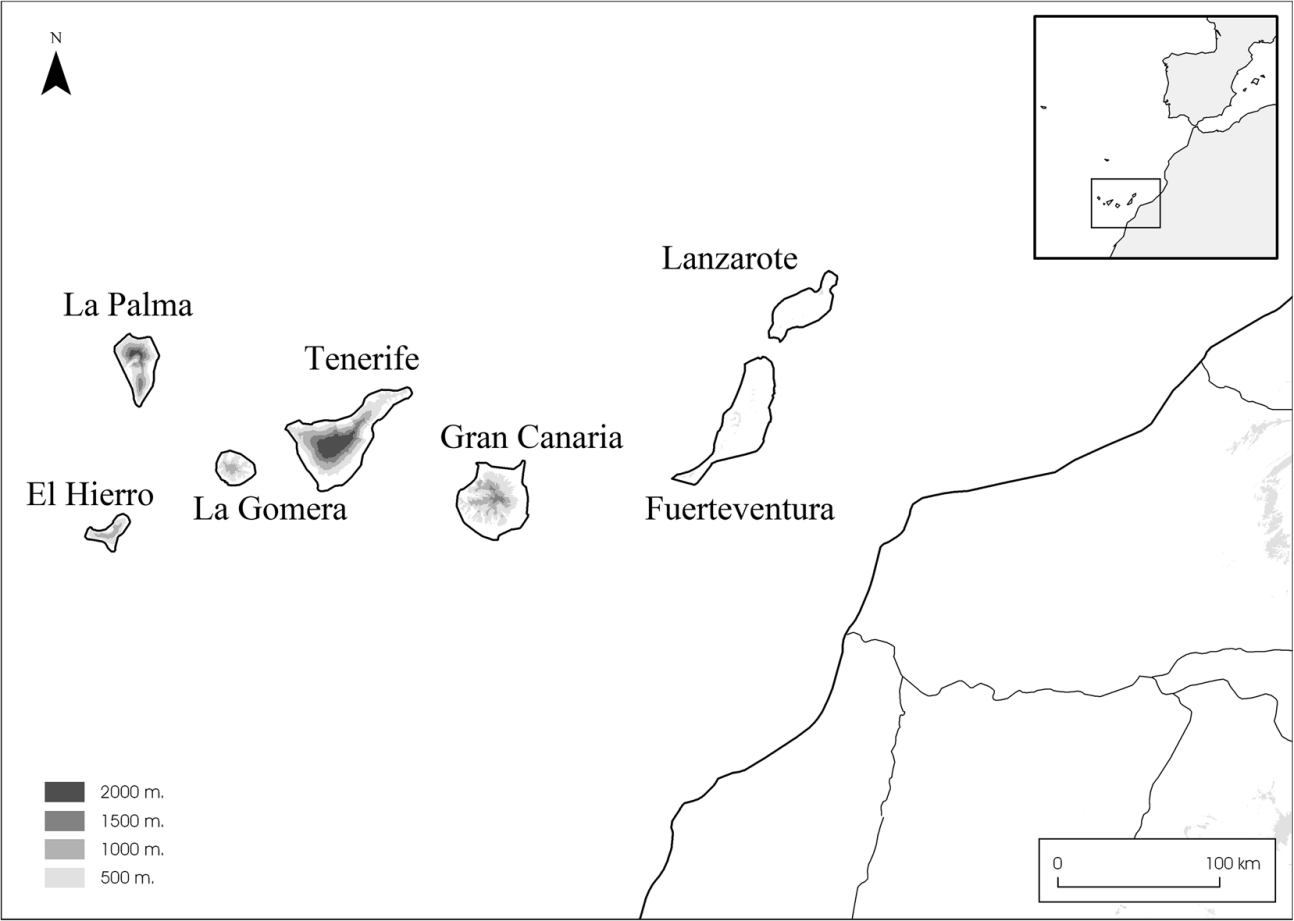
Map of the studied area. Geography of the Canary Islands and location of the Canary archipelago (inset)

The Canary Islands lie some 100 km west of mainland Africa, constituting an Atlantic archipelago with seven major islands (Fig. 1). The islands were settled between the late first millennium BCE and early first millennium CE (Atoche Peña 2013; Atoche Peña and Ramírez Rodríguez 2017; Rodríguez-Rodríguez et al. 2011). The early settlers were likely the Amazigh from North Africa (Fregel et al. 2009a; Fregel et al. 2019; Hagenblad et al. 2017; Maca-Meyer et al. 2003), though there is also evidence of a Roman seasonal settlement on the islet of Lobos in the first century BCE and the first century CE (Del-Arco-Aguilar et al. 2016). After the colonization stage, contact with the mainland ceased. Over time, the populations on the islands also seem to have become isolated from each other (Fregel et al. 2019; Morales et al. 2009). The isolation ended with the rediscovery of the archipelago by European sailors in the fourteenth century (Fregel et al. 2009b; Morales et al. 2009). Henceforth, the term pre-Hispanic will be used to refer to the period between the first colonization of the archipelago and the Castilian conquest of the islands, which lasted the whole fifteenth century. The term Hispanic refers to the period between the Castilian conquest and the present, while the term historical refers to the total colonized period of the islands.

On the Canary Islands, the indigenous people of the pre-Hispanic times developed an agricultural economy with animal husbandry and cultivation of cereal crops (Morales et al. 2009). In particular, barley was a significant crop in their agricultural regime, and it was grown continuously on the islands of Gran Canaria, Tenerife, La Gomera, and El Hierro (Morales et al. 2009; Morales 2010; Morales et al. 2011; Morales et al. 2014). On the island of La Palma, however, initial barley cultivation seems to have been discontinued after the eleventh century and not re-established until after the European colonization (Morales et al. 2013). The pre-Hispanic cultivation on Gran Canaria is well established, with several archaeological sites (up to 14) reporting the presence of barley (Morales et al. 2017; Morales et al. 2018). Historical accounts made by European explorers indicate that at the time of contact, Gran Canaria was the most populated island and had abundant fields devoted to barley production (Boccaccio 1998 [1341]). From Tenerife, archaeological data is sparse but suggest barley as the main crop (Morales et al. 2017). Historical accounts further record the cultivation of barley by the indigenous people, but barley production was not as important as on Gran Canaria. Granaries, such as the ones found on Gran Canaria, have not been attested on Tenerife (De Espinosa 1980 [1594]). Barley cultivation on Lanzarote was reported by the first European visitors (de la Salle 1980 [1404–1419]), but none for Fuerteventura. However, there are currently no archaeological findings supporting barley cultivation on Fuerteventura or Lanzarote. This gap may be due to methodological problems, since systematic sampling and recovering techniques have not been applied in archaeological excavations carried out on these islands.

### Barley Cultivation in Hispanic Times

After the Castilian conquest of the archipelago, the culture of the indigenous society collapsed. Barley cultivation continued in the archipelago in Hispanic times and was reintroduced on La Palma. Data recorded in historical archives indicate that barley kept playing an important role among the cereal crops until the present. In historical documents, the production of barley was measured in bushels (*fanega* in Spanish), a unit of measurement roughly equal to 55.5 liters. In these documents, the agricultural production of Fuerteventura and Lanzarote is well-studied (Santana Pérez 1995). These islands were considered to be the “granaries of the Canaries” because they regularly supplied cereals to the remaining islands of the archipelago. On Lanzarote, data from exports and taxes in the seventeenth century indicate that the production of barley varied between 10,000 and 15,000 bushels (Santana Pérez 2000). Between 1730 and 1736, Lanzarote suffered from devastating volcanic eruptions leading to the destruction of a major part of the fertile land and forcing a large part of the population to leave (Carracedo 2014). Archives from the eighteenth and nineteenth centuries report a larger extension of barley fields on Lanzarote, with the production fluctuating between 43,800 and 140,200 bushels (Gil González et al. 2005).

For Gran Canaria, Hispanic data recovered from the inquisition archives indicate that in 1621 and 1622, the production of barley was 10,000 bushels each year while production of 50,000 bushels is indicated each year in the second half of the eighteenth century (Santana Pérez 1995; Santana Pérez 2000). Tenerife was a leading producer of cereals in the archipelago. Most of the fields were, however, devoted to wheat, while barley was instead imported from Lanzarote and Fuerteventura (Sánchez-Manzano Suárez 2007; Santana Pérez 1995). For instance, from 1800 to 1804, a total of 43,521 bushels of barley were imported from Fuerteventura (Santana Pérez 2004). In 1776, the production of barley on Tenerife was 21,900 bushels, and during the nineteenth century, barley production fluctuated between 22,000 and 24,000 bushels each year (Sánchez-Manzano Suárez 2007).

Today, Gran Canaria is the main island for barley production in the archipelago with more than ten times the output of Tenerife and almost 40 times that of Lanzarote (ISTAC 2019). Genetic comparisons of archaeological barley specimens and contemporary barley from the Canary Islands showed continued cultivation of the same barley gene pool from pre-Hispanic times to the present (Hagenblad et al. 2017). In addition, the genetic characterization of contemporary barley from the archipelago suggested long-term isolation between barley populations from the eastern (Lanzarote and Fuerteventura) and western islands (Gran Canaria, Tenerife, La Gomera, La Palma, and El Hierro). The genetic distinction of barley on Fuerteventura and Lanzarote, on the one hand, and Tenerife on the other, suggests that the barley imports documented during Hispanic times were primarily used for consumption and not cultivation (Hagenblad et al. 2019). There also appeared to be shorter-term isolation between the barley from Gran Canaria and Tenerife (Hagenblad et al. 2017; Hagenblad et al. 2019). The period when this isolation arose on the archipelago is not known.

### Inferring Evolutionary History from Population Genetics

In any given species, genetic material is passed on from parent to offspring. Mutations occurring in the germline become incorporated into the genetic makeup of the lineage and the genetic diversity of a population. In any given population, the current genetic diversity will be a result of the evolutionary processes historically acting on the population. Consequently, information about a population’s evolutionary history will be contained within the genetic diversity of the population (Hodgson and Disotell 2010). When it comes to the study of human history, we need not limit our focus to human genetic diversity (Jones et al. 2013). The genetic studies of domesticated plant species have immense contributions to make to understanding human population and cultural history, especially when such studies are also linked to archaeological investigation.

#### Evolutionary History of Canarian Barley

ABC (approximate Bayesian computation) is a statistical technique that can be used to infer population parameters and choose between different model scenarios (Beaumont 2010). ABC has been used to model colonization scenarios in plants and animals, including humans (e.g., Allen et al. 2020; Francois et al. 2008; Posth et al. 2016). The method has also been used for inferring gene flow and for estimating divergence times and population sizes in phylogeographic models (e.g., Hamilton et al. 2005; Jakobsson et al. 2006; Putnam et al. 2007). In addition, the method is used for modeling scenarios in ecology and epidemiology (Ling et al. 2015; Toni et al. 2008).

In this study, we have sequenced multiple genes in extant landrace populations of barley from the Canary Islands and investigated possibilities and limitations of using ABC modeling to explore the history of Canarian and mainland barley. In particular, we aimed to answer the following questions:

1. When did Canarian barley populations separate from barley populations cultivated on the mainland, and is this separation indicative of the Canary Island colonization event(s)?
2. Compared with the colonization of the Canary Islands, when did barley populations on different islands become isolated from each other?
3. Is there evidence for the pre-Hispanic cultivation of barley on Lanzarote?
4. On what scale was barley historically cultivated on different islands?

## Materials and Methods

### Sampling and Genotyping

Four extant accessions, from Morocco (IG32066), Lanzarote (BGE031112), Gran Canaria (CBT2698), and Tenerife (CBT2609), were chosen to represent the mainland area thought to be closest to the origin of Canarian barley (Hagenblad et al. 2017), an Eastern island of the archipelago, and the two largest western islands of the archipelago, respectively. Based on the results in Hagenblad et al. (2019), accessions with typical genetic diversity of each island (or area), and unlikely to have been recently introduced, were chosen. Genetically typical accessions were not as easily identified for the smaller western islands (Hagenblad et al. 2019), and accessions from these islands were not included in the study. The initial four accessions were later complemented with an additional two accessions from Gran Canaria (CBT2690) and Algeria (INRA11506), chosen on the basis of the genetic representativeness of their respective area of origin. The accessions were obtained from the genebanks IPK (Institut für Pflanzengenetik, Gatersleben, Germany), INIA (Instituto National de Investigación Tecnología Agraria y Alimentaria, Madrid, Spain), CCBAT (Centro de Conservación de la Biodiversidad Agrícola de Tenerife, Tenerife, Spain), and INRA (Institut National de la Recherche Agronomique, Clermond-Ferrand, France) (Table 1). DNA (deoxyribonucleic acid) from 12 individual germinated seeds from each accession was extracted using the DNeasy Plant Mini Kit from Qiagen.

**Table 1:** Studied populations and their origin

Accession number	Origin		Latitude	Longitude
CBT2609	Tenerife	Arico	28.12° N	16.51° W
BGE031112	Lanzarote	Cebada del pais; Teguise	29.06° N	13.56° W
CBT2698	Gran Canaria	Las Palmas	N.A.	N.A.
CBT2690	Gran Canaria	Cebada del pais; Valleseco	28.05° N	15.58° W
IG32066	Morocco	El Zeibe, 18 km from Bab Berret to Tetouan	35.05° N	5.02° W
INRA11506	Algeria	Osiris~us rhyn (Mascara,Vallee du cheliff)	35.38° N	8.38° W

*N.A.*, not available

### PCR and Sequencing

Since optimal flowering time was expected to be similar between the Canary Islands, Northern Africa, and the Fertile Crescent, five flowering time genes (*HvELF3*, *HvGI*, *HvTOC1*, *PpdH1*, and *HvPRR95*), not expected to have experienced strong selection in the study area, were initially chosen. To increase the amount of diversity available for analysis, the five flowering time genes were later supplemented with three additional genes (*Stk*, *Waxy*, and *gapdh*), which were chosen based on their level of diversity in population sequence data deposited at NCBI.

For the accessions CBT2609, CBT2698, BGE031112, and IG32066, fragments from eight genes (*HvELF3*, *HvGI*, *HvTOC1*, *PpdH1*, *HvPRR95*, *Stk*, *Waxy*, and *gapdh*) were amplified using polymerase chain reaction (PCR). The PCR products were between 710 and 843 bp in length and consisted of both exon (approximately 60%) and intron (approximately 40%) sequence. For *PpdH1*, only exon sequence was obtained. For the accessions CBT2690 and INRA11506, only the four most polymorphic genes (*HvELF3*, *HvTOC1*, *Waxy*, and *gapdh*) were PCR-amplified and sequenced because the remaining genes had contributed little or no useful information.

PCR was carried out in 20 μl containing 1 U (units) of DreamTaq polymerase (Thermo Scientific), 1× DreamTaq buffer, 0.1 μM of each primer, and 0.5 μM of each dNTP (Thermo Scientific). PCR was run with an initial 2.30 min at 94 °C followed by 35 cycles of 94 °C for 15 s, locus-specific annealing temperature for 40 s and 72 °C for 40 s, and with a final 72 °C for 10 min. Annealing temperatures were 63 °C for *HvTOC1*; 60 °C for *gapdh*, *Stk5*, and *Waxy*; and 58 °C for *HvELF*, *HvPRR95*, *HvGI*, and *PpdH1.*

Unincorporated nucleotides and primers were removed from the PCR products using 0.014 U/μl Exonuclease I (Thermo Scientific) and 0.0071 U/μl FastAP Thermosensitive Alkaline Phosphatase (Thermo Scientific) incubated at 37 °C for 30 min followed by 5 min at 95 °C. Sequencing was performed by Macrogen Europe in The Netherlands. Resulting sequences are deposited at NCBI under accession numbers MT933205–MT933683.

### DNA Sequence Analysis

Geneious (v 6.0.5) was used to edit and align the DNA sequences (https://www.geneious.com). DNAsp (v 6.12.03) was used to quantify genetic diversity and test for selection with Tajima’s D statistics (Rozas et al. 2017; Tajima 1989). Principal component analysis of the individual sequences was carried out in R (v. 3.1.2) using the *prcomp* command (R Core Team 2018). In the analyses, the presence or absence of each haplotype was used as an independent variable.

#### ABC Modeling

**Fig. 2:**
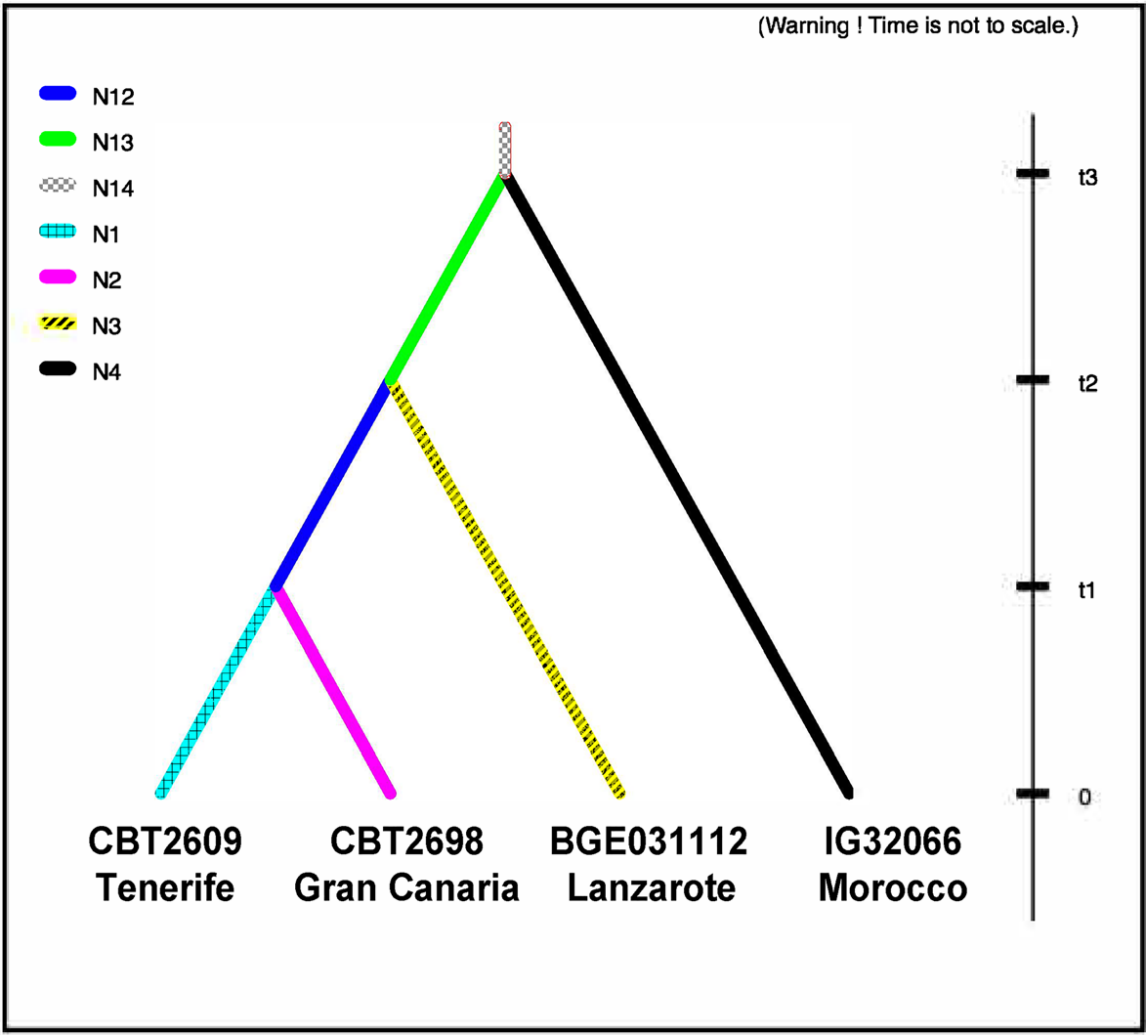
Modeled history of four of the barley populations (different shades of color and patterns denote different population sizes)

Population history was modeled with ABC using DIYABC (v 2.1) (Cornuet et al. 2014). Initially, a model of the history of the four accessions for which sequence data were available from eight loci was set up based on previously described genetic differentiation (Fig. 2; Hagenblad et al. 2017; Hagenblad et al. 2019). Going backward in time, at some time point, t1, the populations from Gran Canaria (CBT2698) and Tenerife (CBT2609) coalesced to a single ancestral population. Further back in time, at time point t2, this ancestral population merged with the population from Lanzarote (BGE031112), and even further back, at t3, the Moroccan population (IG32066) coalesced with the ancestral population of the current Canarian populations. Unique population sizes were allowed for each evolutionary lineage (Fig. 2).

**Fig. 3:**
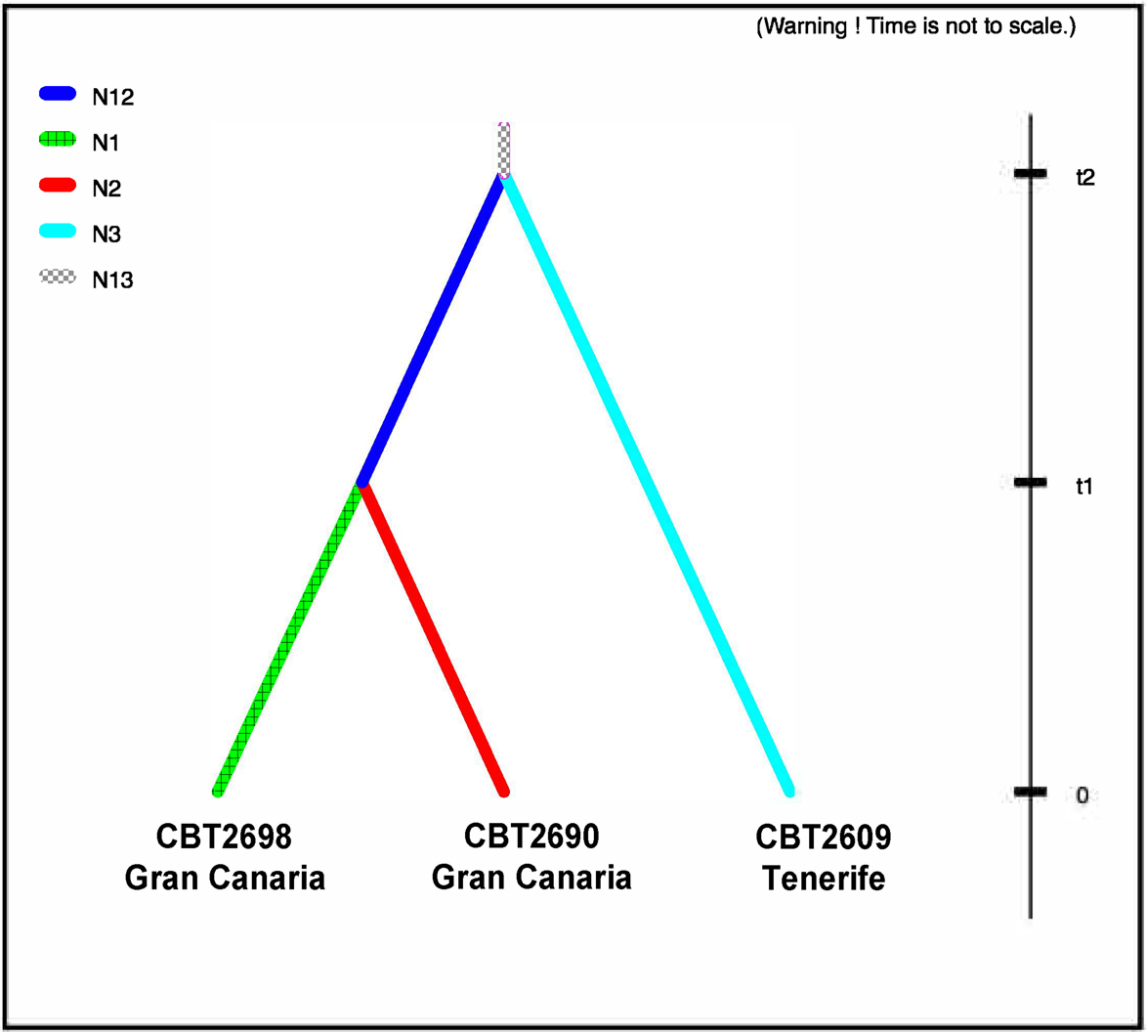
Modeled history of barley populations from Gran Canaria and Tenerife (different shades of color and patterns denote different population sizes)

**Fig. 4:**
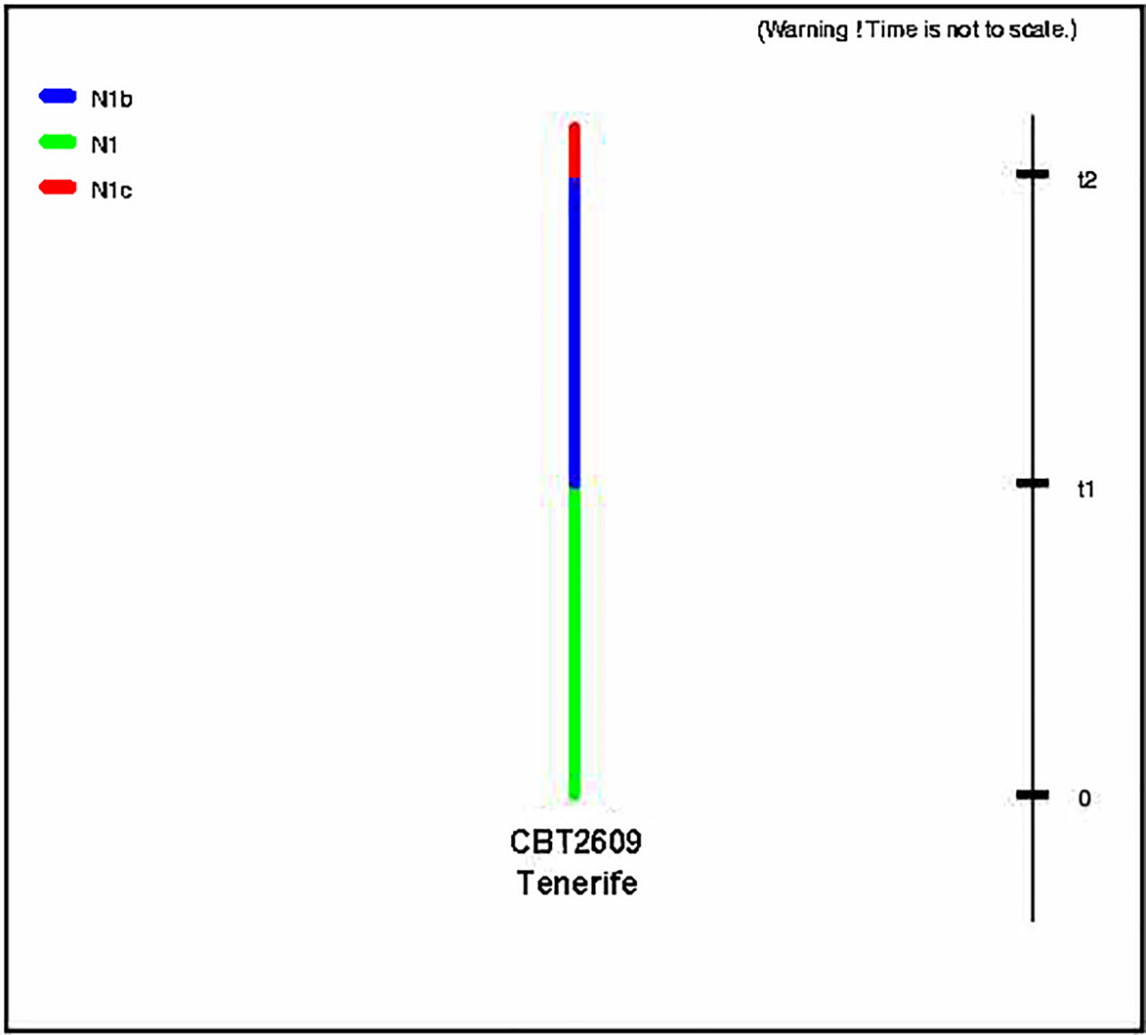
Modeled history of barley population on Tenerife (different shades of color and patterns denote different population sizes)

**Fig. 5:**
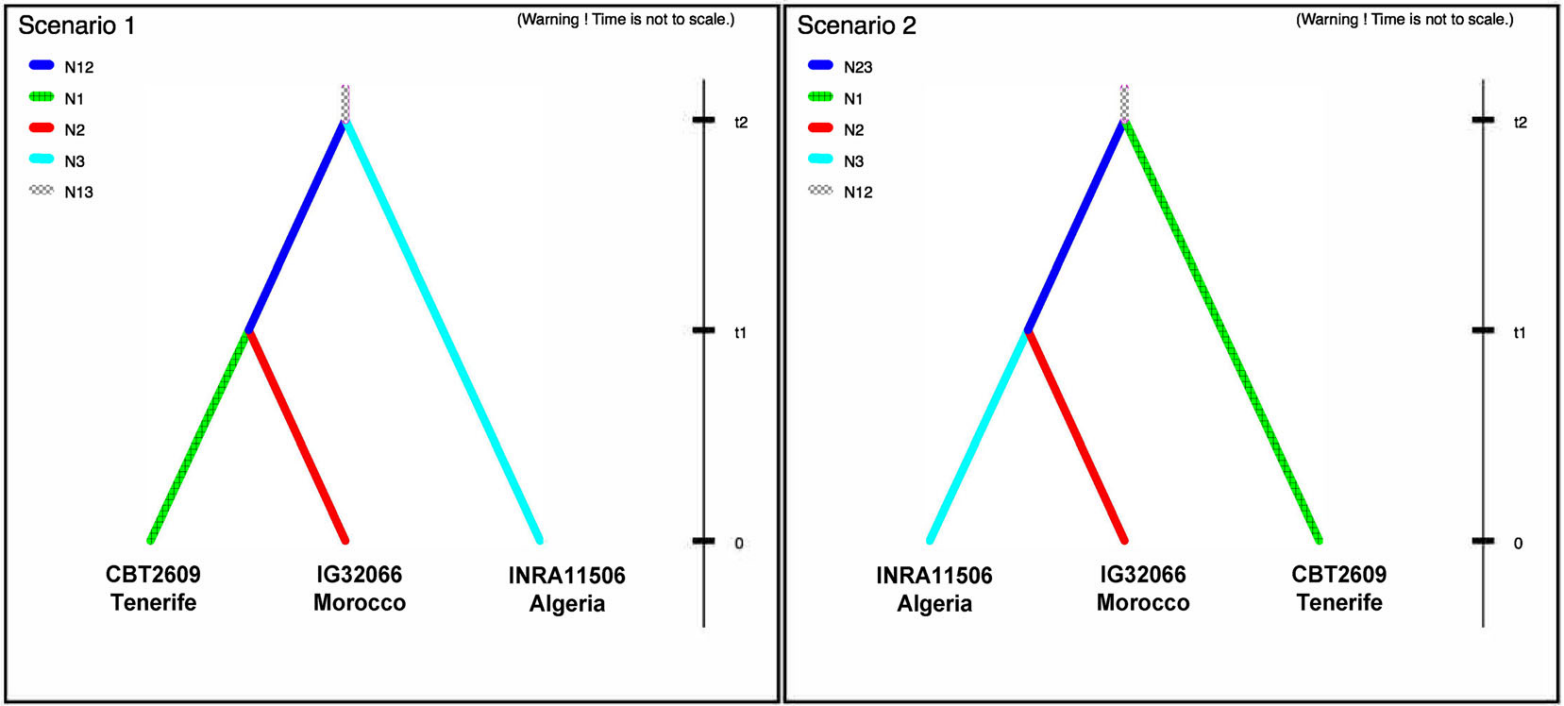
Possible historical scenarios for the Tenerife barley population and two mainland populations

A second dataset, including only the accessions from Gran Canaria (CBT2698 and CBT2690) and Tenerife (CBT2609), was used to model the evolutionary history of the populations according to the scenario shown in Fig. 3. The historical population size of barley on Tenerife was modeled using data from eight genes from only the accession CBT2609. Population size was modeled to have changed at two historical time points, t1 and t2 (Fig. 4). Data from the four most variable genes (*HvELF3*, *HvTOC1*, *Waxy*, and *gapdh*) sequenced in the Tenerife (CBT2609), Moroccan (IG32066), and Algerian (INRA11506) population was used to compare different colonization scenarios (Fig. 5). In scenario 1, Tenerife most recently separated from the geographically closer Moroccan population with an earlier separation from the Algerian population. In scenario 2, the two mainland populations have a common ancestry with an earlier separation from the Tenerife population. A scenario in which Tenerife coalesced first with Algeria is unlikely based on previous results and this is not included in the model (Hagenblad et al. 2017; Hagenblad et al. 2019).

In each simulation of population history, effective population sizes were allowed to vary between 10 and 10,000 individuals, and population splits were allowed to occur between 10 and 10,000 generations before present, with t1 always occurring before t2, and t2 occurring before t3. The mutation rate for the *adh* loci in barley has been determined to 3.5e-9 (Lin et al. 2002). However, initial investigations showed that setting the mean mutation rate to min 1.00e-8 and max 1.00e-6 (rather than the default min 1.00e-9 and max 1.00e-7) resulted in a better fit to the observed data. Hence, this setting was used instead. The percent of invariant sites was set to 20. For the remaining settings, default values were used.

For each scenario sequence, the data from the different fragments were used as observed data (MSS project) with variants within fragments considered to be completely linked. For each population of haplotypes, the number of segregating sites, mean pairwise differences, Tajima’s D, and private segregating sites were calculated; and for each pair of populations, the number of haplotypes, number of segregating sites, and F_ST_ were calculated. For the analysis of the Tenerife population, the mean of numbers of the rarest nucleotide at segregating sites was calculated instead of private segregating sites.

The reliability of the simulated data, compared with that of observed prior and posterior data, was assessed using PCA. The settings above resulted in the observed dataset falling within the range of the first two principal components of both the prior and posterior data of 200,000 simulated data sets. At most, 20% of the summary statistics for the observed data deviated significantly from the summary statistics of the simulated data. In the scenario comparison, posterior probabilities of both scenarios were generated by logistic regression using default settings. Estimates of the time to historical population separation events were adjusted for selfing, according to Nordborg and Donnelly (1997), using a selfing rate for barley set to 98% (Abdel-Ghani et al. 2004).

## Results

### Genetic Diversity and Indications of Selection

Sequencing resulted in a total of 480 sequences. After quality trimming and removal of indels, the resulting sequences were between 577 and 766 nucleotides. The genetic diversity, π, was calculated for eight genes in four populations and four genes in two populations (Table 2). In addition, the number of polymorphic sites, singleton polymorphisms, haplotypes, and private alleles were determined (Online Resource 1). Two of the sequenced genes, *PpdH1* and *Stk*, proved to be completely invariant. *Gapdh* showed high diversity in mainland accessions but was invariant in accessions from the Canary Islands. Within-accession diversity was higher on the mainland (average all genes: 0.0040; genes studied in six accessions: 0.0058) than on the Canary Islands (average all genes: 0.0012; six accessions genes: 0.0020) when looking at all genes or looking only at genes studied in all six accessions (two-sided *t* test, all genes: *p* < 0.01; six accessions genes: *p* < 0.01). For all genes studied in both mainland accessions, total genetic diversity was lower on the Canary Islands than on the mainland (average total diversity mainland: 0.0058; Canary Islands: 0.0024). The number of segregating sites and singletons showed a distribution similar to the genetic diversity (Online Resource 1; two-sided *t* test, all *p* < 0.001), while the number of haplotypes and private alleles were similar for the Canary Islands and the mainland (Online Resource 1; two-sided *t* test, all *p* > 0.05).

**Table 2:** Genetic diversity (*π*) calculated for separate accessions and for sets of accessions

Accession number	Origin	HvELF3	Waxy	HvTOC1	gadph	HvPRR95	Stk	PpdH1	HvGI
BGE031112	Lanzarote	0	0.0025	0.0026	0	0.0002	0	0	0
CBT2609	Tenerife	0.0016	0.0026	0.0076	0	0.0008	0	0	0.0002
CBT2698	Gran Canaria	0.0014	0.0033	0.0017	0	0.0008	0	0	0.0002
CBT2690	Gran Canaria	0.0006	0.0040	0.0035	0				
IG32066	Morocco	0.0014	0.0044	0.0094	0.0096	0.0006	0	0	0.0007
INRA11506	Algeria	0.0016	0.0044	0.0046	0.0108				
All Canarian	Canary Islands	0.0013	0.0030	0.0052	0	0.0007	0	0	0.0006
All mainland	North Africa	0.0014	0.0032	0.0055	0.0101				
All accessions		0.0014	0.0038	0.0060	0.0048	0.0007	0	0	0.0007

In most cases, the sequenced genes showed no sign of selection, population subdivision, or population expansion, when tested using Tajima’s D statistics (Table 3). Population subdivision or expansion is expected to affect loci across the genome, while selection is expected to act on particular genes and linked variants only. Although the genes, in general, tended to have positive rather than negative Tajima’s D values, they were not significantly positive across the genes studied. This suggests that the populations studied experienced negligible amounts of population expansion and subdivision. *HvTOC1* and *gapdh* had significantly positive D values for the mainland populations, separately (*HvTOC1* for both mainland populations and *gapdh* for INRA11506) or together (*gapdh*), indicating a presence of balancing selection in these genes. *Waxy* was the only gene with a significant positive Tajima’s D value for a Canarian accession, CBT2698 (Table 3). All remaining D values were non-significant, suggesting an absence of strong selection acting on the studied genes. The PCA of both the six-population dataset and the four-population dataset visualized not only a certain level of geographic structuring of the genetic diversity but also the presence of shared genetic diversity among populations. Genetic diversity was, in particular, shared among the Canary Islands (Online Resource 2, filled symbols), with mainland accessions being more differentiated (Online Resource 2, open symbols).

**Table 3:** Tajima’s D calculated for separate accessions and sets of accessions (loci deviating significantly from expectations under neutrality are in italics)

Accession number	Origin	HvELF3	Waxy	HvTOC1	gadph	HvPRR95	Stk	PpdH1	HvGI
BGE031112	Lanzarote	N.A.	0.8279	- 0.3129	N.A.	- 1.1405	N.A.	N.A.	N.A.
CBT2609	Tenerife	0.6721	1.0291	0.8756	N.A.	1.4862	N.A.	N.A.	-1.1405
CBT2698	Gran Canaria	1.8912	2.2762 *	- 1.5273	N.A.	1.4862	N.A.	N.A.	1.8912
CBT2690	Gran Canaria	- 1.6293	1.7412	0.8678	N.A.				
IG32066	Morocco	1.8912	0.9724	2.0525 *	1,9500	0.6714	N.A.	N.A.	1.8922
INRA11506	Algeria	0.8723	1.7412	2.2171 *	2.7188 **				
All Canarian	Canary Islands	0.9615	0.2944	0.6443	N.A.	1.4254	N.A.	N.A.	1.3188
All mainland	North Africa	1.0127	1.1434	1.5288	3.9678 ***				
All accessions		0.5626	0.0523	1.4044	0.4895	0.3127	N.A.	N.A.	1.6340

*N.A.*, not available. *p < 0.05; **p < 0.01; ***p < 0.001

### Historical Population Sizes

Approximate Bayesian coalescent modeling was used to estimate historical population parameters under different scenarios. The posterior distributions of the time estimates were, as for the population size estimates, overlapping (Online Resource 3). In a four-accession model, the population size of Lanzarote (BGE031112) had the smallest mean estimate (mean effective population size, *N*_e_ = 2,200; 95 % interval 200–8,000). The mean estimated population size of Gran Canaria (CBT2698) was smaller (mean *N*_e_ = 3,300; 95 % interval 400–9,000) than that of Tenerife (CBT2609, mean *N*_e_ = 7,200; 95% interval 240–9,900), and that of the ancestral population of Gran Canaria and Tenerife, i.e., the population eventually giving rise to the barley cultivated on both Gran Canaria and Tenerife (mean *N*_e_ = 6,500; 95% interval 1,200–9,900). The ancestral mean population size of all Canarian populations was marginally larger than that of Lanzarote (mean *N*_e_ = 2,900; 95% interval 100–9,000). The estimated mean population of Morocco was about the same size as that of Tenerife (IG32066, mean *N*_e_ = 6,800; 95% interval 2,400–9,800).

To rule out the possibility that the small mean estimate of the population size of the Gran Canaria accession was caused by an outlier population factor, the four most variable genes (*HvELF3*, *HvTOC1*, *Waxy*, and *gapdh*) were sequenced in an additional population from Gran Canaria, and historical population sizes were estimated from a dataset of only the Gran Canaria accessions (CBT2698 and CBT2690) and the accession from Tenerife (CBT2609). The mean population size estimates of CBT2698 and CBT2609, when based on the subset of most variable genes, were similar (CBT2698 mean *N*_e_ 3,600; 95% interval 400–9,200; CBT2609 mean *N*_e_ 6,800; 95% interval 2,000–9,900) to those based on the four-accession dataset (with data from eight genes). This suggests that leaving out the four least variable genes did not result in a significant loss of power when estimating population sizes. The population size estimates for CBT2690, from Gran Canaria, were more similar to those of the other Gran Canaria population (CBT2698) than to the Tenerife population (CBT2609; CBT2690 mean *N*_e_ 4,500; 95% interval 600–9,500), albeit overlapping. The mean estimate of the ancestral population size for the Gran Canaria populations was also lower than that of the Tenerife CBT2609 (mean *N*_e_ 5,000; 95% interval 400–9,700), suggesting a generally smaller historical population size on Gran Canaria than on Tenerife.

The analysis shows variation in the historical Tenerife barley population size (0–t1 mean *N*_e_ 500; 95% interval 100–4,600; t1–t2 mean *N*_e_ 5,600; 95% interval 300–9,800). The changes in population size were estimated to most likely have occurred before the colonization of the island in the fourth through the second-century BCE (mean estimate of t1: 8,100 years ago, 95% interval 400–17,400 years ago) (Galván Santos et al. 1999) and could probably not be attributed to differences in pre-Hispanic and Hispanic scales of cultivation (before and after the fifteenth century). It should be noted, however, that posterior distributions suggested limited power to accurately estimate any parameter but the most recent population size (Online Resource 3).

### Timing of Population Separations

Estimates of the timing of separation of populations were obtained from the four-accession model (Fig. 2). The posterior distributions of the time estimates were, as for the population size estimates, overlapping in all cases, though with distributions narrowly distributed around the means (Online Resource 3). After adjusting for the high selfing rate of barley (*s* = 0.98, see Abdel-Ghani et al. 2004 and references therein), the Gran Canaria (CBT2698) and Tenerife (CBT2609) populations were estimated to have separated from each other some 1,200 years ago (mean estimate of t1 in Fig. 2; 95% interval 100–3,800 years ago). Lanzarote (BGE31112) was estimated to have separated from the other Canarian populations some 1,800 years ago (mean estimate of t2 in Fig. 2; 95% interval 300–6,500 years ago), while the Canarian populations separated from the mainland about 2,400 years ago (mean estimate of t3 in Fig. 2; 95% interval 300–11,600 years ago). Estimates of separation times, based on only the four genes available from the two Gran Canaria accessions (CBT2690 and CBT2698) and Tenerife (CBT2609), were higher than those from the four-accession dataset (CBT2690 and CBT2698 mean estimate 2,500 years ago, 95% interval 200–8,800 years ago; Gran Canaria and Tenerife mean estimate 3,100, 95% interval 200–13,900) and had wider distributions of the posterior estimates (Online Resource 3).

The data from the four most variable genes sequenced in the Tenerife, Moroccan, and Algerian populations was used to compare different colonization scenarios. A scenario where the Tenerife population separated from the mainland populations before the mainland populations separated from each other (scenario 2 in Fig. 5) had the highest posterior probability (scenario 1, 0.2818, CI 0.0296–0.5341; scenario 2, 0.7182, CI 0.4659–0.9704; Online Resource 4), suggesting that the choice of mainland population had little effect on the modeling of the Canarian population history.

## Discussion

The accessions used in this study were all obtained from genebanks. Concerns have been raised over how well genebank material represents the originally deposited accessions with respect to the amount and authenticity of the genetic diversity (Chebotar et al. 2002; Chebotar et al. 2003; Hagenblad et al. 2012; Parzies et al. 2000). The accessions used in this study were chosen among a large number of Canarian and Moroccan accessions previously screened to be genetically representative of their area of origin (Hagenblad et al. 2017; Hagenblad et al. 2019). Three of the accessions (CBT2609 collected in 2012, CBT2690 in 2006, and CBT2698 in 2011) have gone through a single regeneration in the gene bank. They are, therefore, unlikely to have experienced changes in their genetic composition during genebank conservation. Of the remaining accessions, INRA11506 was collected in 1921, but there is no collection date available for BGE031112 and IG32066. We note that BGE031112 is more diverse than more recently collected accessions from the same island and that IG32066 is more diverse than the nineteenth-century historical seeds from Algeria and Tunisia, but INRA11506 shows less genetic diversity than the historical Algerian seeds (Hagenblad et al. 2017). Taken together, we consider the majority of the studied accessions to be sufficiently good representatives of barley originally cultivated at the different areas of origin.

Eight different genes were chosen for sequencing. Since selection will affect the coalescence history of a sample, flowering time genes were initially chosen. Photoperiod and climate were expected to be similar between the Fertile Crescent, North Africa, and the Canary Islands, and these factors were expected to have imposed little selection on flowering time genes. In some studies, however, functional and putatively functional genetic diversity in flowering time genes has been shown to exist among accessions longitudinally separated along the same latitude (Aslan et al. 2015; Cockram et al. 2011; Jones et al. 2008; Jones et al. 2016). Such variation has been attributed both to selection during domestication and to recent crop movements (Cockram et al. 2011; Lister et al. 2009). It is, however, worth noting that diversity among accessions from the same latitude *per se* is not evidence of selection but will also occur during neutral evolution. The expectation of limited selection on the flowering time genes proved to be true insofar that the diversity distribution of these genes showed little signs of selection, in particular on the Canary Islands. The flowering time genes, however, had relatively little genetic diversity, which could be a consequence of purifying, but here undetected, selection. The flowering time genes were complemented with additional, more variable, genes and these, likewise, showed little evidence of selection. The data was consequently considered acceptable for evaluation with ABC modeling. Although deviations from neutrality can affect the coalescence history, the effects on the different populations studied here should be similar. Comparisons among populations should thus be possible, although the actual size and time estimates should be interpreted with caution. Where Tajima’s D deviated from the expectation under neutrality, primarily in mainland accessions, values were positive, suggesting the presence of population subdivision or balancing selection.

It should be noted that the estimates of population sizes came with rather substantial estimate distributions between the upper and lower quartile. The wide estimate distributions are partially inherent in the coalescent model and depend on the amount of diversity studied. A larger number of loci, though not necessarily a larger number of individuals, would have provided more power for modeling the population history and would likely have resulted in a narrower estimate distribution. Furthermore, the relationship between the estimated effective population size (*N*_e_) and the cultivated census size is not clear. It, therefore, seems more prudent to compare relative differences between the sizes of different populations rather than to take the mean values reported above at face value. It can be noted that estimates based on a larger number of genes typically had a narrower distribution. It is thus likely that more reliable estimates, and hence a more certain and detailed picture of the history of barley cultivation on the Canary Islands, can be obtained with sequence data from a larger set of genes. Such a data set could, for example, be obtained with high-throughput sequencing strategies.

Population size estimates for the different islands were largely congruent for the four-accession model and the Gran Canaria – Tenerife model. They pointed to a historically larger population size on Tenerife than on Gran Canaria, in contrast with present-day barley production (ISTAC 2019). In pre-Hispanic times, Gran Canaria was the more populated of the two and with a larger agricultural production (Mederos Martín 2019; Morales et al. 2017). The archaeology of Gran Canaria is characterized by the presence of large settlements, as well as communal granaries where surplus production was stored (Morales et al. 2018). The complexity of the indigenous society of Gran Canaria has been linked to a large agricultural production, based on barley, that could feed the entire population (Velasco Vázquez 1999). Analyses of the bone and dental status of skeletal remains from pre-Hispanic individuals on Gran Canaria indicate a diet based on cereals, confirming the link between the existence of large populations and the practice of agriculture (Arnay-De-La-Rosa et al. 2010; Delgado Darias 2009; Velasco Vázquez 1999).

At multiple archaeological sites on Gran Canaria, both settlements and granaries, barley remains have been recorded along with hard wheat, but with barley always being the more abundant cereal (Morales et al. 2017). Nevertheless, this points to barley being cultivated at a larger scale on Gran Canaria than Tenerife in pre-Hispanic times, contrary to the population sizes estimated from the ABC model. It should be pointed out, however, that systematic sampling of seeds in archaeological excavations has been more extensive on Gran Canaria than on Tenerife. On Tenerife, evidence comes from three cave sites at which seeds were separated by hand-sieving, a less efficient technique that is not suitable for recovering small seeds and plant fragments such as cereal chaff (Morales et al. 2017). In contrast, systematic sampling and flotation of sediments have been carried out extensively on Gran Canaria at more than ten sites providing a more accurate record of the pre-Hispanic crops (Morales et al. 2017; Morales et al. 2018). Therefore, the available botanical data is biased, and no definitive conclusions should be drawn about this issue. Archaeological finds and evidence recorded in historical texts in the fifteenth and sixteenth centuries suggest that the indigenous people of Tenerife were pastoralists, highly mobile, and lacking large villages (Arnay-De-La-Rosa et al. 2010). More studies are needed to obtain reliable information on the significance of pre-Hispanic agriculture on this island.

Since the Castilian conquest, human population sizes and cereal production have been larger on Tenerife than on Gran Canaria, which could have contributed to the larger barley population size estimates obtained for Tenerife. It is also possible that differing amounts of inter-island seed exchange have influenced population size estimates of the two islands (Santana Pérez 2000). We note, however, that previous characterizations of barley diversity on Gran Canaria and Tenerife showed similar distributions of the intra-island genetic diversity (Hagenblad et al. 2019). Additionally, past fluctuations in the scale of barley cultivation, on Gran Canaria, Tenerife, or both islands, undetected in the archaeological record, could have contributed to the estimates of a larger barley population size on Tenerife. Major crop failures on one of the islands, but not the other, could have contributed to the reduction of the population size estimated with ABC modeling. Tenerife has a somewhat higher annual rainfall and may have been less susceptible to crop failure due to drought. Nevertheless, systematic sampling and analyses of the seed remains in archaeological sites on Tenerife are still undeveloped, and further investigations of pre-Hispanic barley cultivation might shed additional light on the question of inter-island differences in barley population sizes (Sánchez-Benítez et al. 2017).

Although the barley production on Lanzarote during Hispanic times has, at times, been substantial (Gil González et al. 2005), the barley population size found on Lanzarote is smaller than on the western islands; this agrees with contemporary data (ISTAC 2019) and historical cultivation records (Santana Pérez 2000). The Historical-period large agricultural production on Lanzarote may well have been offset by a drastic population bottleneck following the 1730–1736 volcanic eruptions. In addition, comparisons of the population size estimates from the different Canary Islands with population size estimates for the mainland suggest that barley cultivation has been as extensive on Tenerife as in the Moroccan population sampled. This raises intriguing questions concerning the historical scale of cultivation and extent of seed exchange in northern Africa.

The scale of barley cultivation on Tenerife has varied historically, yet attempts to model differences in population size met with little success. Differences were detected, but the changes predated the colonization of the Canary Islands. It is possible that a larger dataset, with respect to the number of loci and individuals, could yield sufficient information to understand recent changes in the scale of cultivation. It should be noted that the analysis of genomic data, where the two homologous chromosomes of an outbred individual were analyzed with the PSMC (pairwise sequentially Markovian coalescent) model, primarily detected population size changes in the range between 10 Kya and 10 Mya (Li and Durbin 2011). With multiple sequentially Markovian coalescent (MSMC) analyses of multiple genomes, population changes prior to the past 2,000 years, corresponding to 80–100 generations, could be detected (Schiffels and Durbin 2014). With such a resolution, changes in the scale of cultivation during both pre- and Hispanic times could potentially be detected in Canarian barley.

As with population size, estimates of the time of population separation came with substantial estimate distributions, partly as a consequence of the limited data set. The time estimates were, however, more narrowly distributed around the mean than the size estimates, allowing for some interesting (even if tentative) conclusions. The comparison of the estimate from the four-accession model and that of the dataset, including only accessions from Gran Canaria and Tenerife, yielded rather different time estimates, although the estimates for the four-accession model were all included in the range for Gran Canaria and Tenerife. This is probably a result of the small number of loci (four) for which sequence data was available for the Gran Canarian accession CBT2690. The narrower range of estimates obtained from the larger number of loci supports the expectation that increasing the number of loci studied will be necessary to obtain a more precise time estimate.

The comparison of the different historical scenarios for the Tenerife population and two mainland populations, based on data from only the four genes, strongly suggested that the choice of mainland barley population had little effect in this study. Although the colonization of the Canary Islands may not predate the separation of the barley cultivated in Morocco and Algeria, the evolutionary lineage from which barley was brought to the Canary Islands seems to have split from the one leading to the populations cultivated in Morocco and Algeria before the two mainland populations became distinct. It is not known to what extent historical seed exchange occurred in the region, but seed exchange was encouraged in some medieval documents, including those by the Andalusian Arabic author Ibn Al Awwam (Zadoks 2013). There is the possibility of historical seed exchange in North Africa after the colonization of the Canary Islands.

Despite the uncertainties in the estimates of the separation of barley populations, it can be noted that the mean estimates from the four-accession model fit surprisingly well with what is known about the colonization of the Canary Islands from the archaeological records. The mean separation age of the Canarian populations from the mainland population (2,400 years ago) is a little older than the currently favored colonization date of the Canary Islands—100 BCE to 100 CE (Atoche Peña 2013; Fregel et al. 2019; Rodríguez-Rodríguez et al. 2011). However, it should be noted that the Moroccan barley population is not necessarily the the ancestor of the Canarian barley populations and that the separation of the two may well predate the actual colonization date by a few hundreds of years. It should also be noted that the mean estimates obtained from the four-accession model do not contradict the previously suggested older archaeologically derived colonization dates for the archipelago (Atoche Peña and Ramírez Rodríguez 2017; Galván Santos et al. 1999; Navarro Mederos 1983; Zöller et al. 2003).

Interestingly, the estimate of the separation time of the barley populations from Lanzarote and the western islands, 1,800 years ago, is very close to the currently favored colonization date of the Canary Islands. Archaeological research has led to the conclusion that the Canary Islands were colonized between the late first millennium BCE and the early first millennium CE (Atoche Peña 2013; Atoche Peña and Ramírez Rodríguez 2017; Rodríguez-Rodríguez et al. 2011). The genetic analyses of barley from Lanzarote confirm the continued cultivation of the same local barley on Lanzarote from the colonization of the Canary Islands through the 1730–1736 volcanic eruptions (de la Salle 1404-19/1980; Hagenblad et al. 2017; Hagenblad et al. 2019). They also tentatively suggest that the eastern and western islands quickly became isolated from each other or that seed exchange was discontinued between them (Fregel et al. 2019; Morales et al. 2009). At the time of European arrival in the fourteenth and fifteenth centuries, there was no maritime contact among the islands, and the populations on each island spoke different but mutually intelligible dialects (Navarro Mederos 1983). Archaeological data also points to a lack of exchanges and connections within the archipelago (Morales et al. 2017). An absence of seed exchange likely allowed barley on the eastern islands to adapt to the particular arid climatic conditions on the islands (Gil González 2011).

In contrast, the estimates of the separation of the Gran Canaria and Tenerife barley populations, 1,200 years ago, hint at the tantalizing possibility of continued contact and seed exchange between the islands for several centuries after the initial colonization of the Canary Islands. The two islands are clearly visible from each other on a clear day, and the possibilities for traveling between the two must have been evident to the indigenous people. There is, however, currently no archaeological evidence of exchanges between Gran Canaria and Tenerife in pre-Hispanic times. It is also possible that the time estimates for the separation between the Gran Canaria and Tenerife barley populations are a consequence of later colonization of these more westerly islands. The population separation estimates are congruent with a multi-step colonization model where Tenerife was colonized from Gran Canaria several hundred years after contact had ceased with Lanzarote. Indeed, accelerator mass spectrometry on short-life specimens has yielded earlier dates from Lanzarote (100–300 cal CE; Arco-Aguilar and Ramirez-Rodriguez 2011; Atoche Peña and Ramírez Rodríguez 2011) than from Gran Canaria (400-500 cal CE; Velasco Vázquez 2015) and Tenerife (660–880 cal CE; Morales et al. 2017). Either way, the ABC modeling suggests an end of seed exchange between Gran Canaria and Tenerife, for whatever reason, several centuries before the advent of European sailors.

## Conclusions

To conclude, the results of the ABC modeling support several existing hypotheses and archaeological inferences concerning the agricultural history of the Canary Islands. It also pointed to areas where additional investigations may be beneficial. The ABC modeling resulted in time estimates that fit well with archaeological conclusions about the timeline for the initial settlement of the Canary Islands. Since the first human colonization of the islands (100 BCE–100 CE), the same population of barley has been continuously cultivated on Lanzarote. However, soon after the initial colonization, Lanzarote appears to have been isolated from the more western islands. Seed exchange between Gran Canaria and Tenerife seems to have ceased several centuries after the islands’ isolation from Lanzarote, but well before the islands were conquered by Europeans. Alternatively, the colonization of the two islands was separated by several centuries. Although Gran Canaria is currently responsible for the majority of barley production, cultivation seems to have been more common on Tenerife in the pre-Hispanic time. It is possible that barley was cultivated on Tenerife on a scale comparable to that of the Moroccan mountains.

## Electronic Supplementary Material

ESM 1 Number of haplotypes, private alleles, segregating sites, and singleton detected in the sequenced genes in the different populations, for the mainland populations, all Canarian populations, and Western Canarian populations (XLSX 12 kb)

ESM 2 (a). Results for six-population dataset. Results of principal component analysis of sequence data. Blue filled squares denote CBT2698, red filled circles denote CBT2690, green filled triangles denote CBT2609, brown diamonds denote BGE03112, purple open circles denote IG32066, and orange open squares denote INRA11506 respectively. (b). Results for the four-population dataset. Results of principal component analysis of sequence data. Blue filled squares denote CBT2698, green filled triangles denote CBT2609, brown diamonds denote BGE03112, and purple open circles denote IG32066 (PDF 468 kb)

ESM 3 Prior and posterior distributions for the four-population dataset (a); the Gran Canaria and Tenerife dataset (b); and the four-population dataset (c) (PDF 775 kb)

ESM 4 Posterior probabilities for two scenarios for the relationship between barley from Tenerife, Morocco, and Algeria: (a) Direct estimates; (b) Logistic regression estimates (PDF 63 kb)

## Data Availability

The sequences generated in this study are available at NCBI under accession numbers MT933205–MT933683.
