# Supplementary material for: An Evolutionary Approach to the History of Barley (Hordeum vulgare) Cultivation in the Canary Islands
Source: Afr Archaeol Rev. 2020 Oct 2;37(4):579–95. doi: 10.1007/s10437-020-09415-5 (PMC7677147; doi:10.1007/s10437-020-09415-5)
Supplement: Supplementary file 2 — (a). Results for six-population dataset. Results of principal component analysis of sequence data. Blue filled squares denote CBT2698, red filled circles denote CBT2690, green filled triangles denote CBT2609, brown diamonds denote BGE03112, purple open circles denote IG32066, and orange open squares denote INRA11506 respectively. (b). Results for the four-population dataset. Results of principal component analysis of sequence data. Blue filled squares denote CBT2698, green filled triangles denote CBT2609, brown diamonds denote BGE03112, and purple open circles denote IG32066 (PDF 468 kb) [file 10437_2020_9415_MOESM2_ESM.pdf]

a)

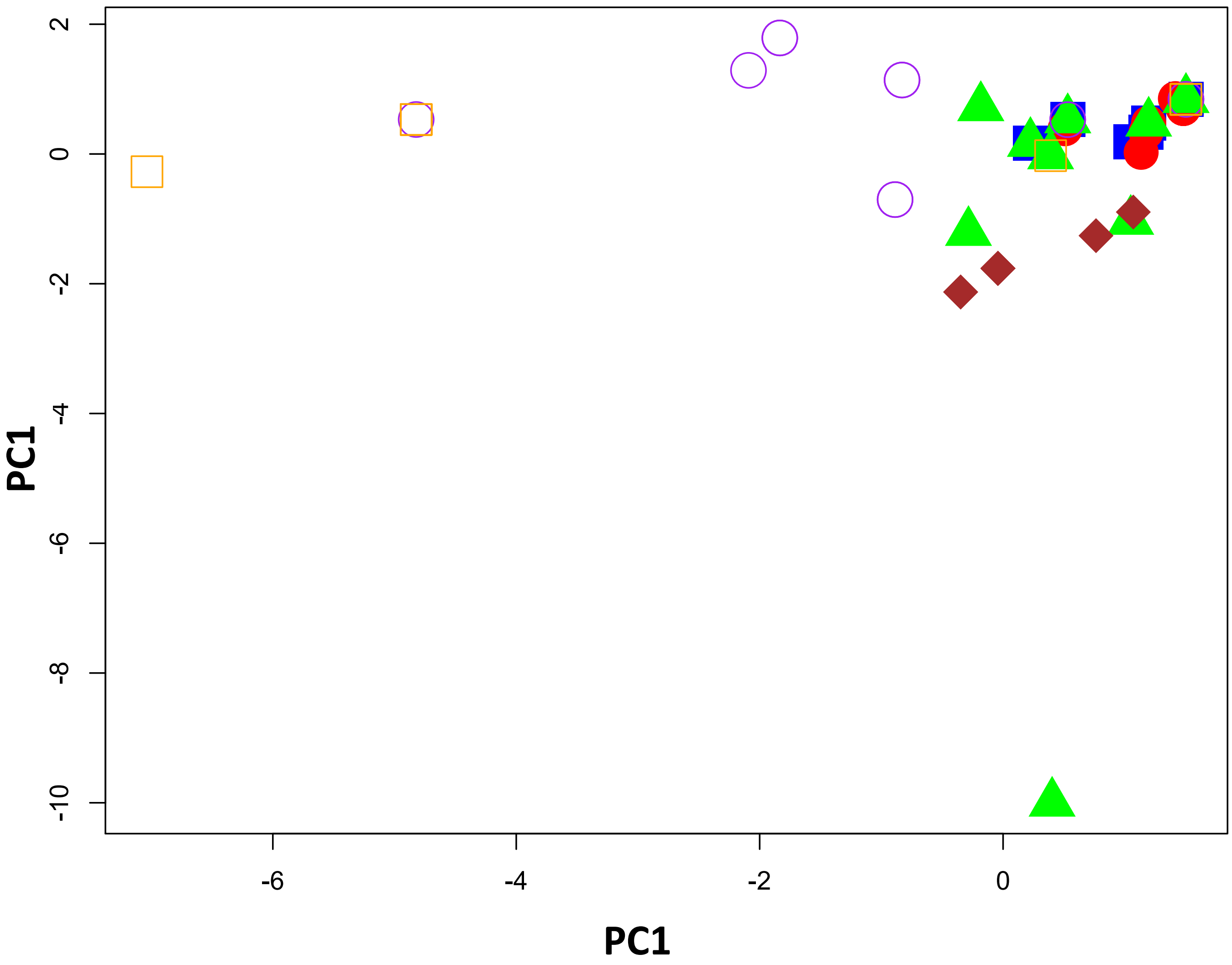

Online Resource 2:
(a). Results for six-population dataset. Results of principal component analysis of sequence data. Blue filled squares denote CBT2698, red filled circles denote CBT2690, green filled triangles denote CBT2609, brown diamonds denote BGE03112, purple open circles denote IG32066, and orange open squares denote INRA11506 respectively.

b)

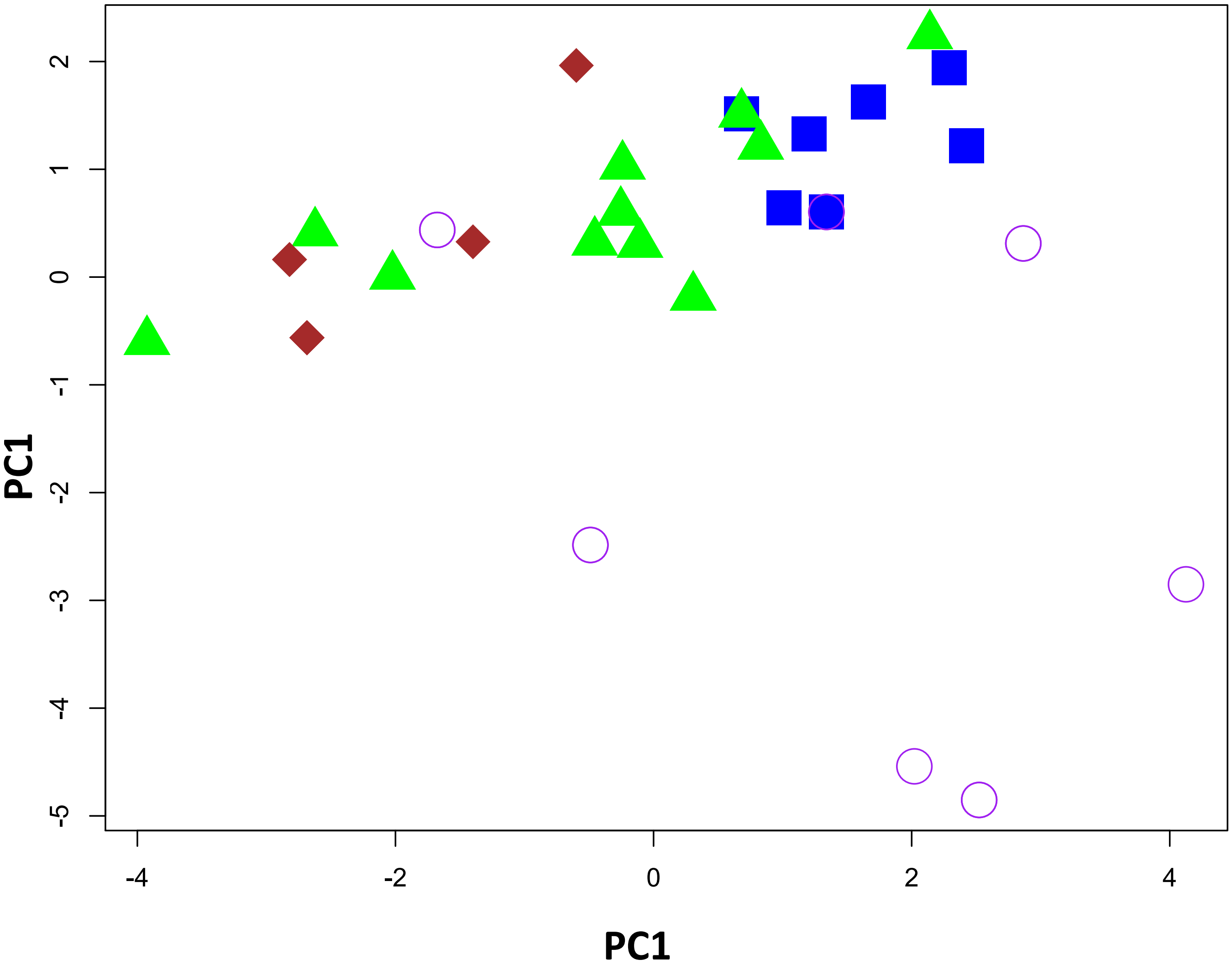

Online Resource 2:
(b). Results for the four-population dataset. Results of principal component analysis of sequence data. Blue filled squares denote CBT2698, green filled triangles denote CBT2609, brown diamonds denote BGE03112, and purple open circles denote IG32066.
