# Supplementary figures and images for: An Evolutionary Approach to the History of Barley (Hordeum vulgare) Cultivation in the Canary Islands
Source: Afr Archaeol Rev. 2020 Oct 2;37(4):579–95. doi: 10.1007/s10437-020-09415-5 (PMC7677147; doi:10.1007/s10437-020-09415-5)

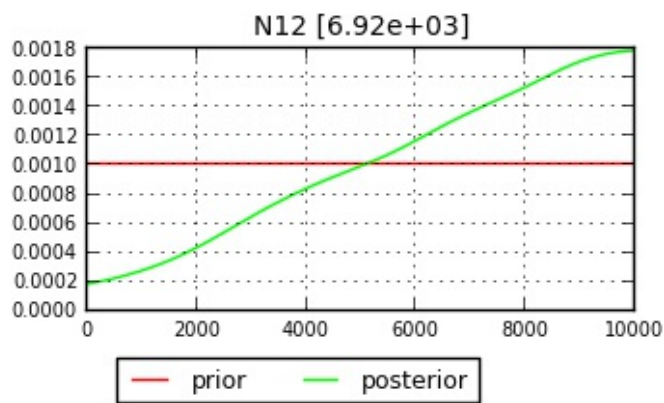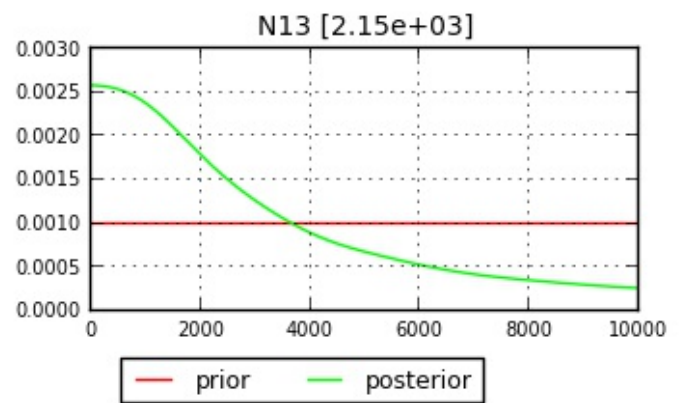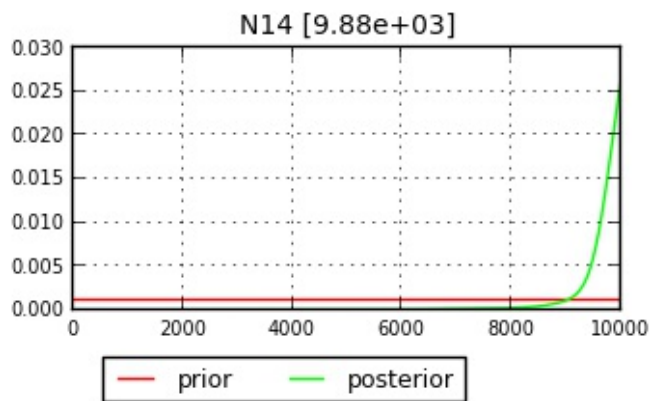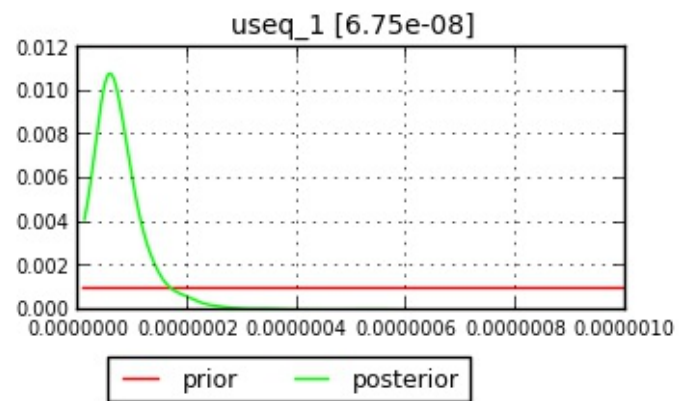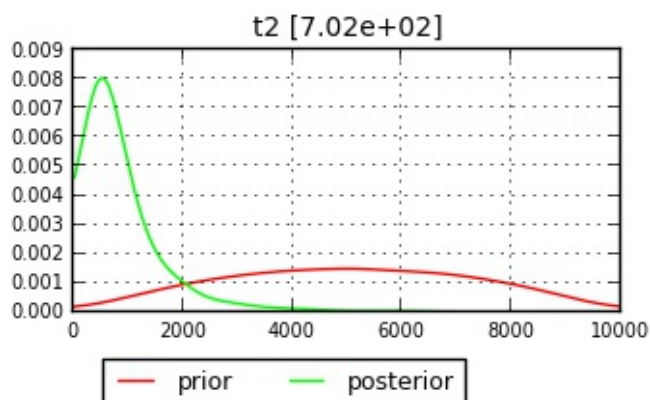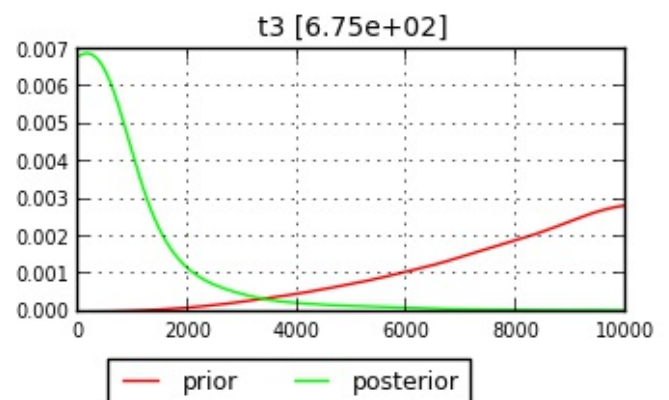

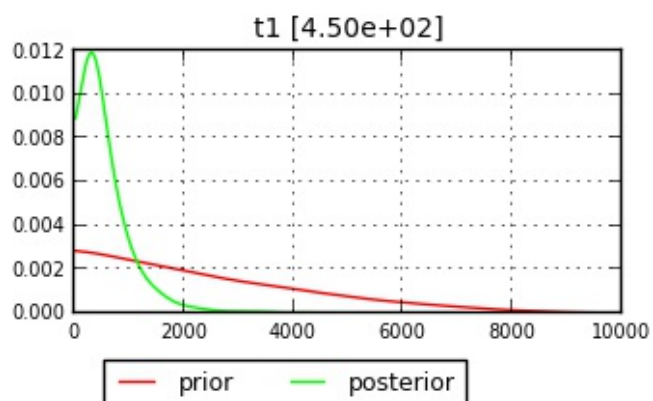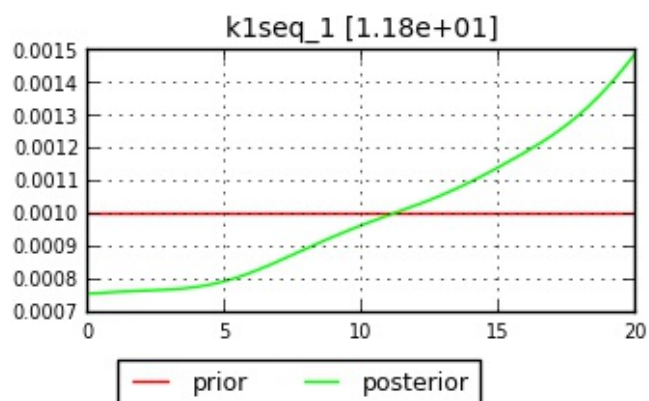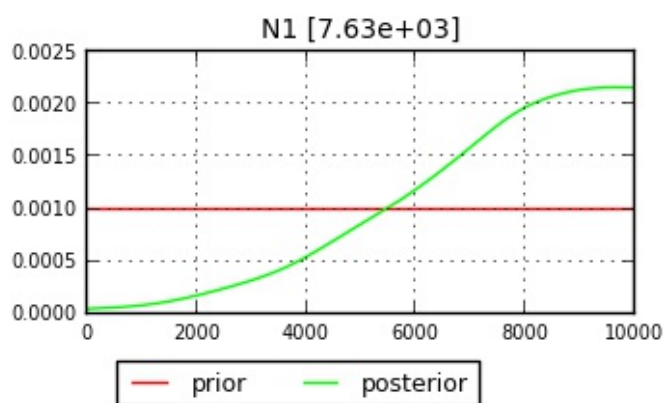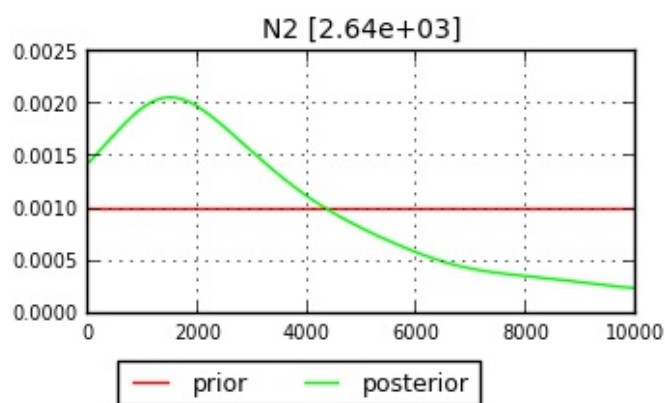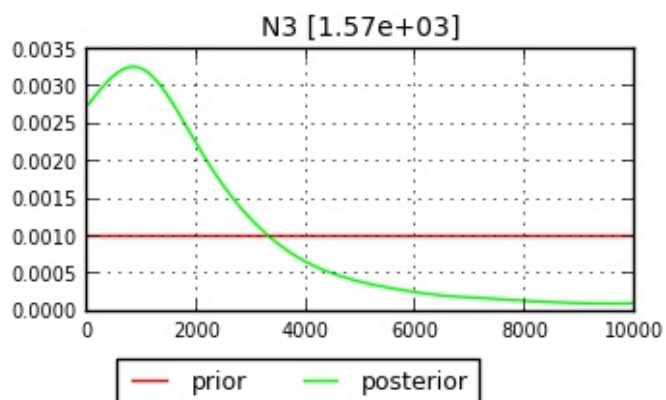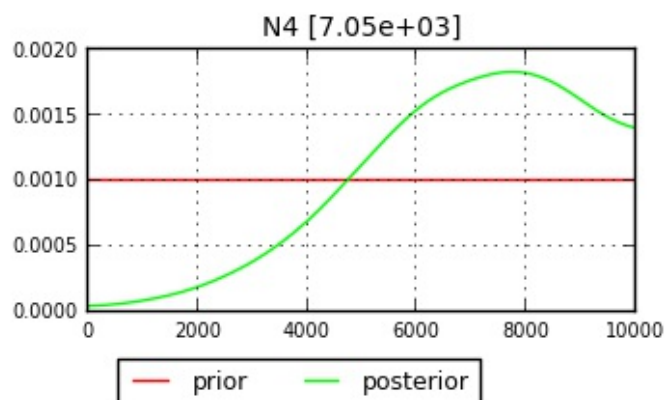

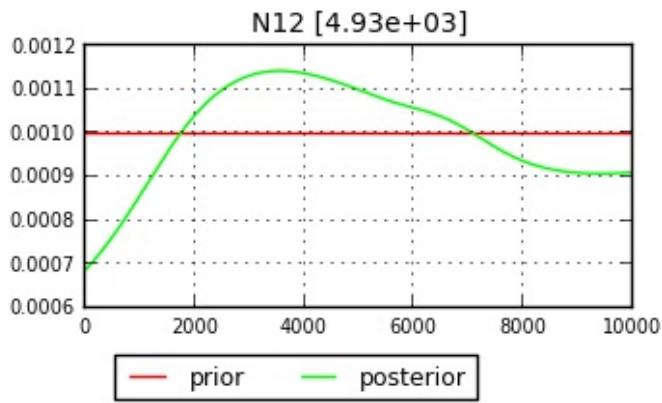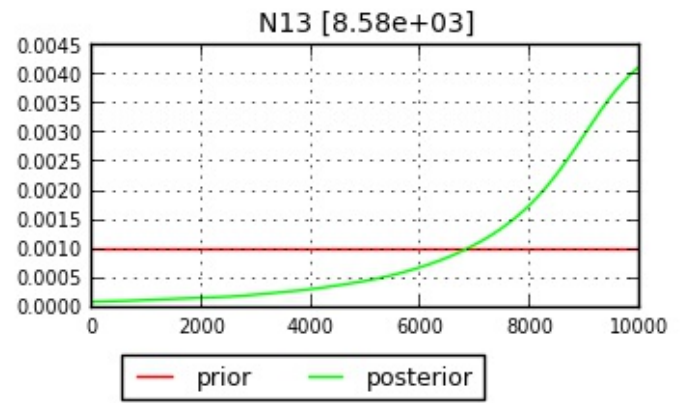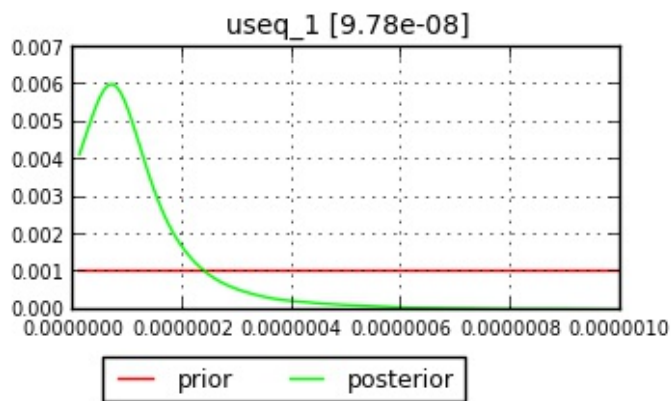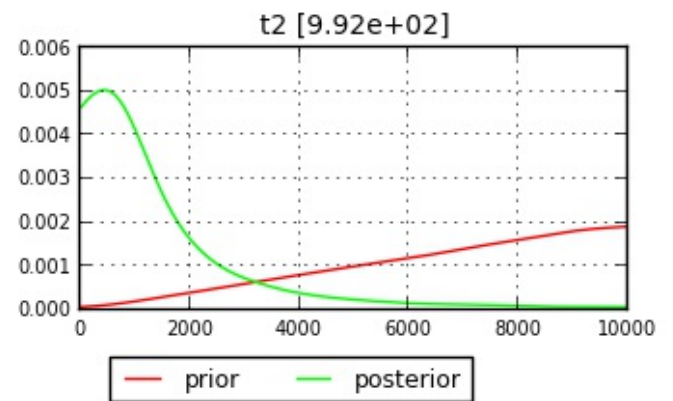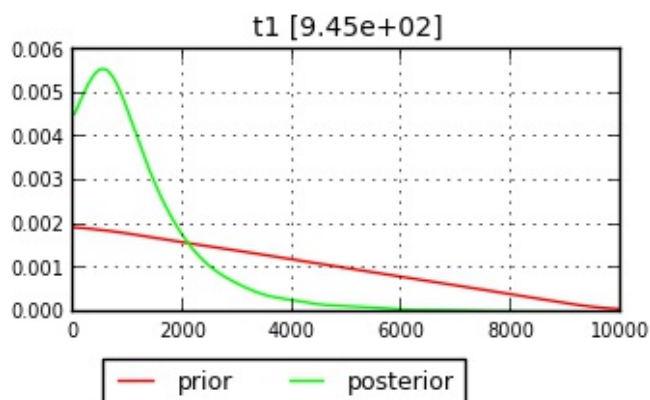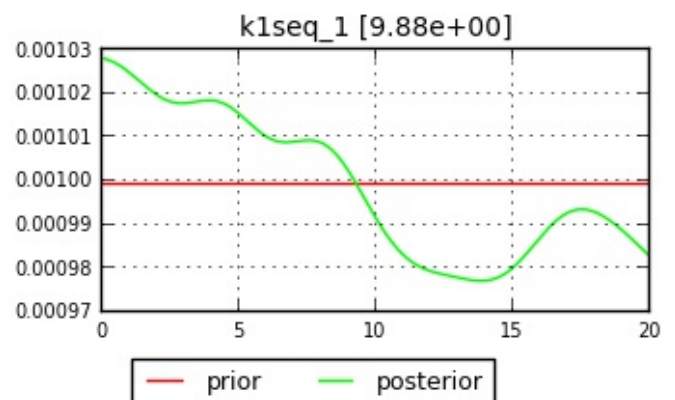

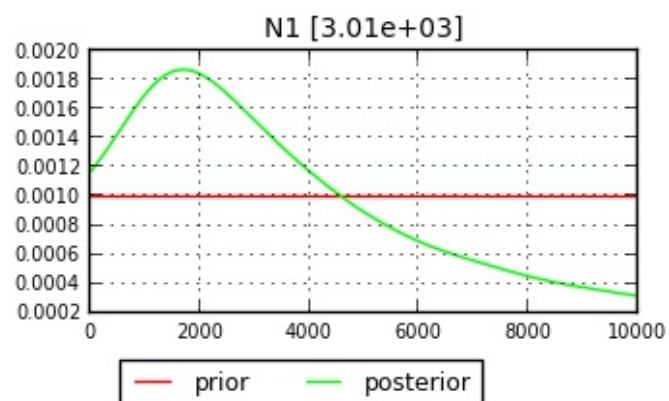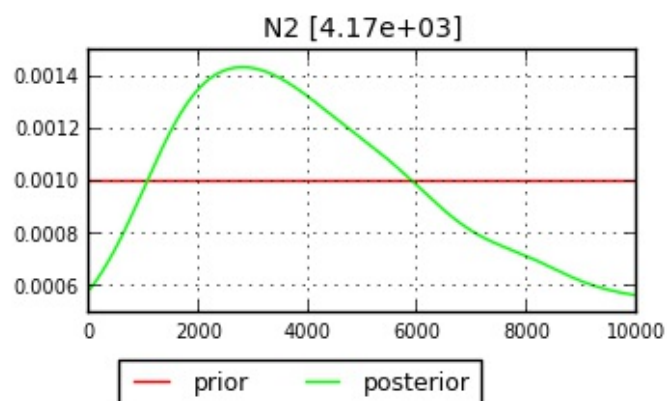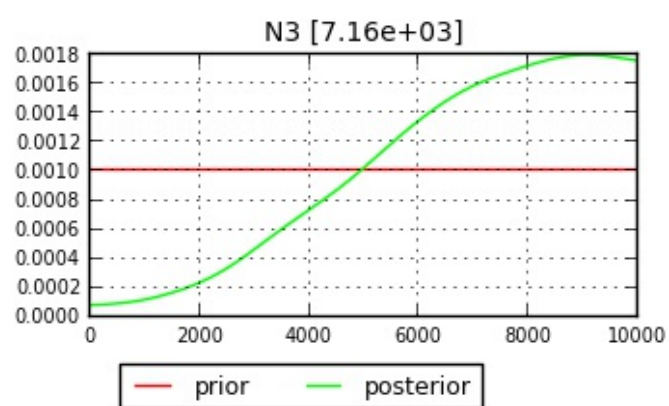

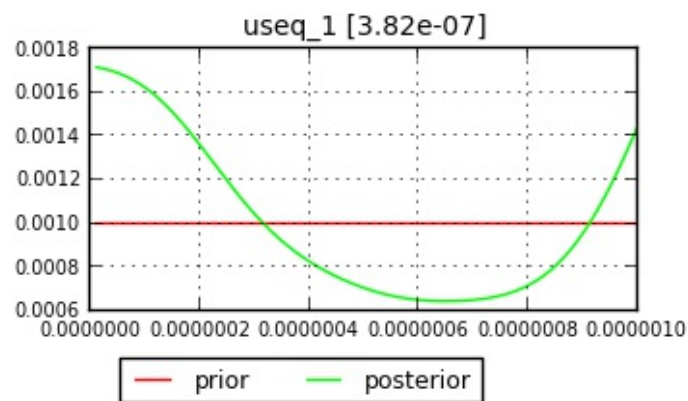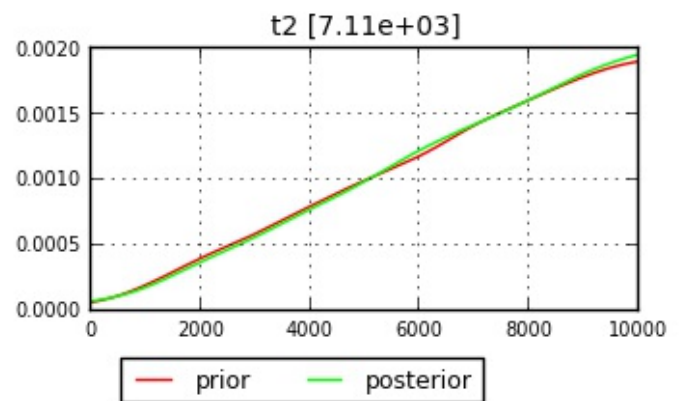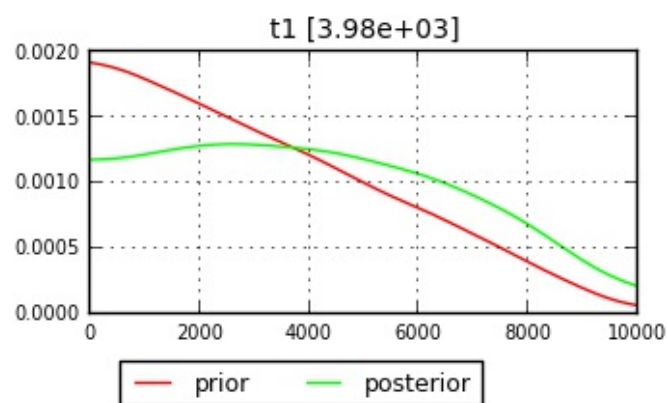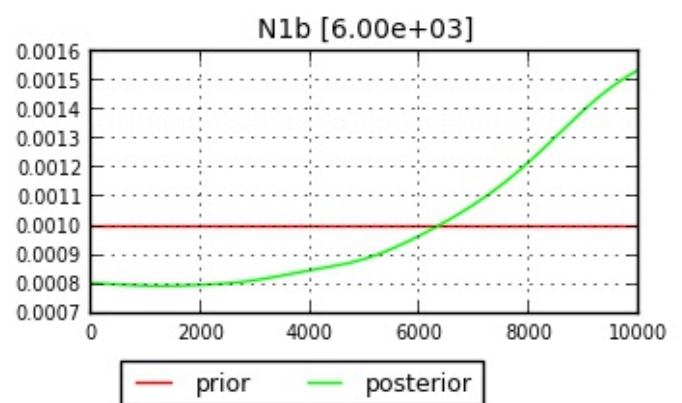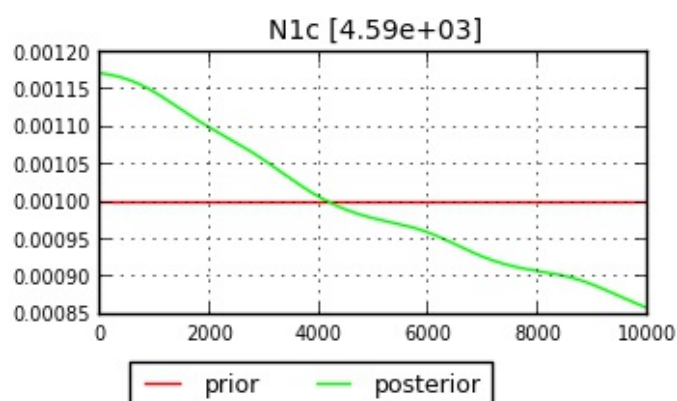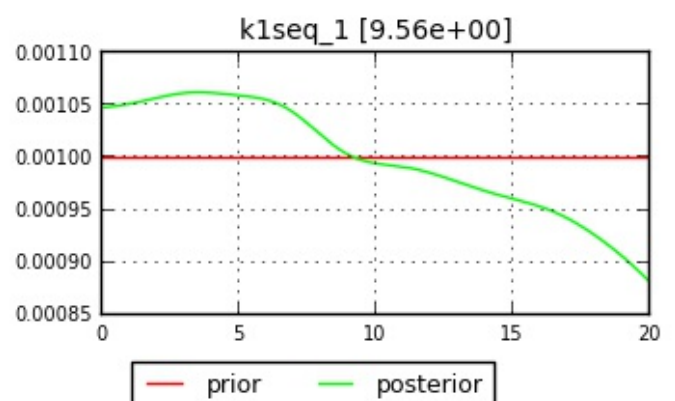

Supplement: Supplementary file 3 — Prior and posterior distributions for the four-population dataset (a); the Gran Canaria and Tenerife dataset (b); and the four-population dataset (c) (PDF 775 kb) [file 10437_2020_9415_MOESM3_ESM.pdf]
