# Supplementary material for: An Evolutionary Approach to the History of Barley (Hordeum vulgare) Cultivation in the Canary Islands
Source: Afr Archaeol Rev. 2020 Oct 2;37(4):579–95. doi: 10.1007/s10437-020-09415-5 (PMC7677147; doi:10.1007/s10437-020-09415-5)
Supplement: Supplementary file 4 — Posterior probabilities for two scenarios for the relationship between barley from Tenerife, Morocco, and Algeria: (a) Direct estimates; (b) Logistic regression estimates (PDF 63 kb) [file 10437_2020_9415_MOESM4_ESM.pdf]

a)

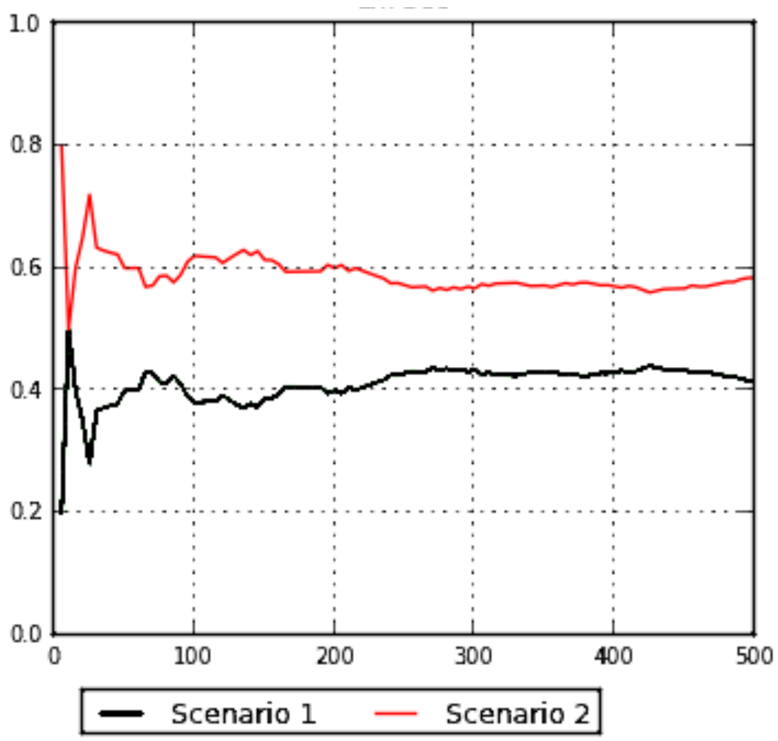

b

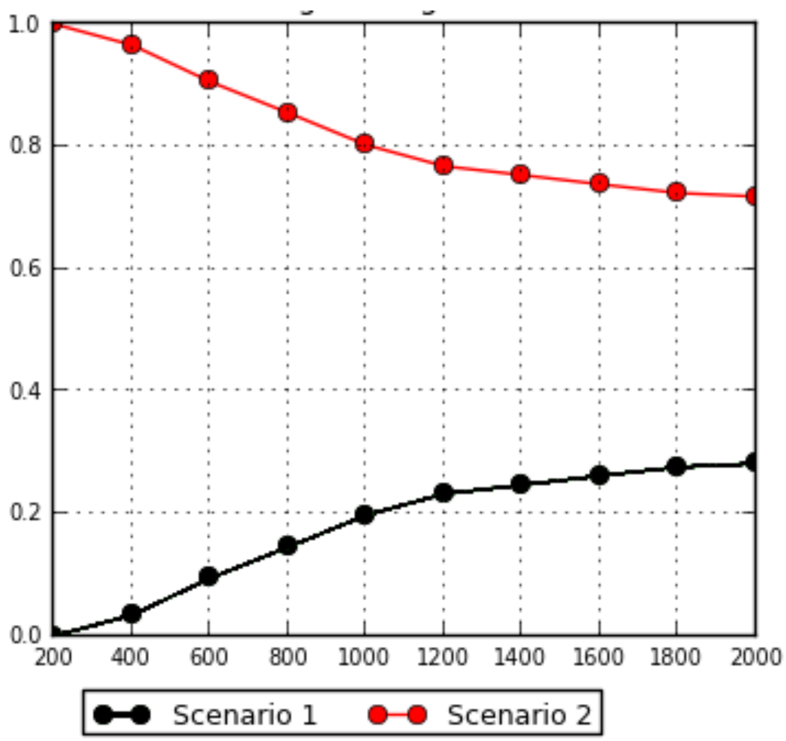

Online Resource 4. Posterior probabilities for two scenarios for the relationship between barley from tenerife, Morocco and Algeria a) Direct estimates b) Logistic regression estimates
